# Quantitative Structure–Retention Relationship Analysis of Polycyclic Aromatic Compounds in Ultra-High Performance Chromatography

**DOI:** 10.3390/molecules28073218

**Published:** 2023-04-04

**Authors:** Fabrizio Ruggieri, Alessandra Biancolillo, Angelo Antonio D’Archivio, Francesca Di Donato, Martina Foschi, Maria Anna Maggi, Claudia Quattrociocchi

**Affiliations:** 1 Dipartimento di Scienze Fisiche e Chimiche, Università degli Studi dell’Aquila, Via Vetoio, 67100 Coppito, Italy; 2 Hortus Novus, Via Campo Sportivo 2, 67050 Canistro, Italy

**Keywords:** polycyclic aromatic hydrocarbons, quantitative structure–retention relationship, artificial neural network, Partial Least Squares Regression, ultra-high performance liquid chromatography

## Abstract

A comparative quantitative structure–retention relationship (QSRR) study was carried out to predict the retention time of polycyclic aromatic hydrocarbons (PAHs) using molecular descriptors. The molecular descriptors were generated by the software Dragon and employed to build QSRR models. The effect of chromatographic parameters, such as flow rate, temperature, and gradient time, was also considered. An artificial neural network (ANN) and Partial Least Squares Regression (PLS-R) were used to investigate the correlation between the retention time, taken as the response, and the predictors. Six descriptors were selected by the genetic algorithm for the development of the ANN model: the molecular weight (MW); ring descriptor types *nCIR* and *nR10*; radial distribution functions *RDF090u* and *RDF030m;* and the 3D-MoRSE descriptor *Mor07u*. The most significant descriptors in the PLS-R model were MW, *RDF110u*, *Mor20u*, *Mor26u*, and *Mor30u*; edge adjacency indice *SM09_AEA (dm)*; 3D matrix-based descriptor *SpPosA_RG*; and the GETAWAY descriptor *H7u*. The built models were used to predict the retention of three analytes not included in the calibration set. Taking into account the statistical parameter RMSE for the prediction set (0.433 and 0.077 for the PLS-R and ANN models, respectively), the study confirmed that QSRR models, associated with chromatographic parameters, are better described by nonlinear methods.

## 1. Introduction

Polycyclic aromatic hydrocarbons (PAHs) are global contaminants and are defined as being composed of two or more fused aromatic rings. PAHs are released into the environment from a variety of anthropogenic sources, including the burning of fossil fuels, coal, and wood, and petrochemical processes like cracking [1,2,3]. They are emitted by incomplete combustion of organic materials in internal combustion engines, in electricity and heat generation, and in the metal and asphalt pavement industries. Moreover, they can be produced naturally during forest fires and volcanic activity [4,5,6,7]. PAHs have been identified in most of the abiotic and biotic compartments because they are recalcitrant to chemical and biological degradation [8,9,10]. PAHs are considered hazardous environmental contaminants because they exhibit mutagenic and carcinogenic proprieties [11,12]. Due to their remarkable toxicological properties, the international supervisory authorities have defined PAHs as priority contaminants [13]. Therefore, PAHs are frequently analyzed in environmental compartments and biological samples. The US Environmental Protection Agency (EPA) has defined a list of 16 unsubstituted PAHs as priority pollutants [14,15]. Gas chromatography is a primary method for analyzing low-molecular-weight PAHs, but it is not optimally suited for the higher-molecular-weight analytes (≥C_24_-PAH) due to their low vapor pressures [16,17]. Nevertheless, most studies reported in the literature that applied QSRR methods for this class of compounds involved gas chromatography [18,19,20]. 

Quantitative structure–retention relationships (QSRRs) represent the theoretical description of chromatographic retention behavior using physicochemical properties derived from the chemical structure of analytes and from the effect of chromatographic conditions [21,22,23,24,25,26,27]. A method of optimization to represent the correct geometry of each analyte is required to provide data for the calculations of molecular descriptors. Once the geometry is optimized, the molecular descriptors of the analytes can be calculated. These models help to predict the retention of the analytes and, subsequently, to find the optimal analytical conditions in the domain of applicability of the developed models. In recent studies, several linear and nonlinear models based on the QSRRs approach have been developed to predict the retention time from the chemical and structural properties of the compounds under several eluent compositions [28,29,30,31,32]. 

**Figure 1:**
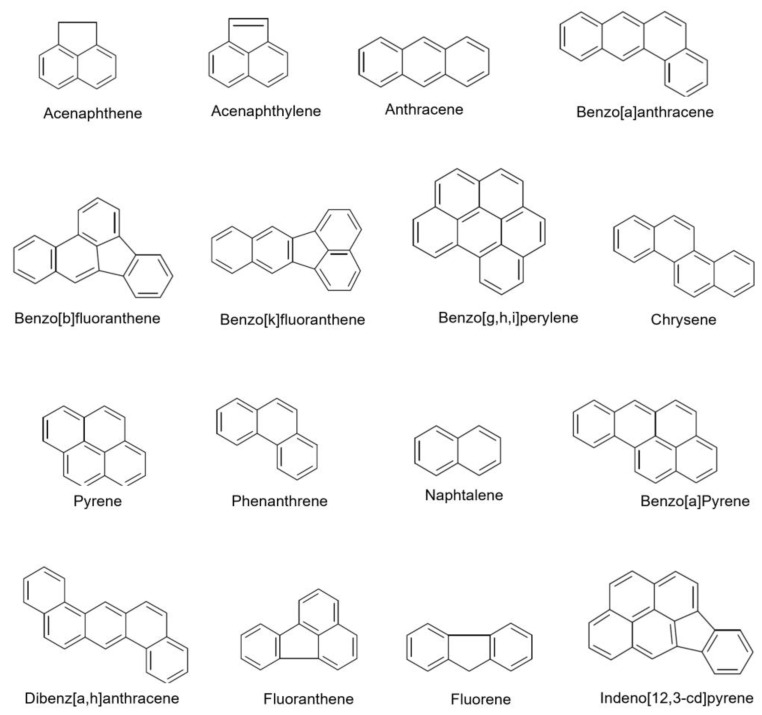
Chemical structures of the analyzed PAHs.

The PAHs chosen for this study are a selection of compounds that are commonly found in environmental samples and have been identified as priority pollutants by the EPA. These compounds were chosen based on their structural range, which ranges from 2 to 6 condensed rings. This range includes some of the most toxic and carcinogenic PAHs, such as benzo[a]pyrene, as well as less toxic compounds, such as naphthalene, as reported in Figure 1. The number of molecules used in a QSRR study may depend on several factors, including the structural homogeneity of the molecules being studied [33,34,35,36,37,38]. 

In this study, ultra-high-performance liquid chromatography (UHPLC), equipped with diode array detection, was used to detect sixteen PAHs. UHPLC has been increasingly adopted in chemical laboratories as a result of its high resolution, high speed, and solvent economy. UHPLC methods involve a reduced time of analysis and result in an improved chromatographic resolution and reproducibility, compared with a classical HPLC method [39,40,41,42,43]. All these advantages provide a more complete knowledge of the samples analyzed and make it possible to obtain a large amount of data in a very short time. Analysis of the retention mechanisms for a homologous series of compounds can provide valuable information about the physical and chemical properties of the compounds and their interactions with the stationary phase in the chromatography column. Since the retention phenomenon depends on molecular properties and experimental chromatographic conditions, different QSRR models were built considering both molecular descriptors and different chromatographic parameters.

The software Dragon was used to calculate 4885 molecular descriptors [44]. Due to the huge number of variables, selecting the most explanatory ones was required. The variables representing redundant or useless information must be recognized and rejected to achieve adequate models. Genetic algorithms (GAs) are some of the widely used variable selection methods in this area [45]. A GA is a stochastic process to solve optimization problems defined by fitness criteria applying Darwin’s evolution hypothesis and different functions such as cross-over and mutation [46,47,48]. Multiple linear regression (MLR), applied to GA, was employed to select molecular descriptors to be used in successive prediction models. The retention time of the analytes was collected under gradient elution conditions by varying the column temperature, the mobile phase flow rate, and the run time. The models were built starting from the retention times of the analytes contained in the training set in the domain of the chromatographic conditions and were successively applied to predict the retention of the external compounds. Different chemometric approaches were used, namely, Partial Least Squares Regression (PLS-R) and backpropagation artificial neural network (ANN). For the PLS-R, the dimensionality was reduced following different criteria and introducing, by construction, new orthogonal latent variables (LV) [43], linear combinations of the original ones [49,50]. LVs were calculated to explain most of the covariance between the original predictor data matrix and the response. Molecular descriptors were also elaborate with the ANN [51,52,53], guaranteeing a wide range of input types, the possibility to apply nonlinear functions, higher resistance against outliers, and improved flexibility compared to linear techniques [54]. The outcomes were compared with the GA-ANN approach; one of the main advantages of ANNs is that despite the chromatographic retention being a complex process and its dependency on the molecular descriptors often not being well-established, ANNs can represent a suitable tool for handling it. Several applications of QSRR models in HPLC analysis are reported in the literature [22,24,29]; nevertheless, at present, very few studies report QSRR for the prediction of the retention time in UHPLC [28,55].

## 2. Results and Discussion

### 2.1. Variable Selection by Genetic Algorithm

In this work, GA-MLR analysis was performed using the program package V-PARVUS 2010 [56]. This program generates a random population of 100 chromosomes that are subjected to crossover and mutation. In the crossover process, two mating chromosomes exchange their genetic material according to the “uniform crossover technique”, in which for each gene, a random number determines if it will undergo crossover. The mutation is due to a random change in the value of a gene based on a very low probability selected (here, 1%). Elitism, the number of the best chromosomes of each generation passing unchanged to the next one, is set to 2% to avoid the loss of highly predictive models. Evolution of the initial population is carried out for 50 evolution cycles, or it is stopped after 5 cycles if no improvement of R^2^ loo-cv (coefficient of determination in leave-one-out cross-validation) is observed. GA-MLR variable selection was carried out following the criteria listed in Table 1.

Using GA-MLR analysis, we identified a six-dimensional multilinear model; the selected solute descriptors are collected in Table 2.

### 2.2. QSRR-ANN Model

**Figure 2:**
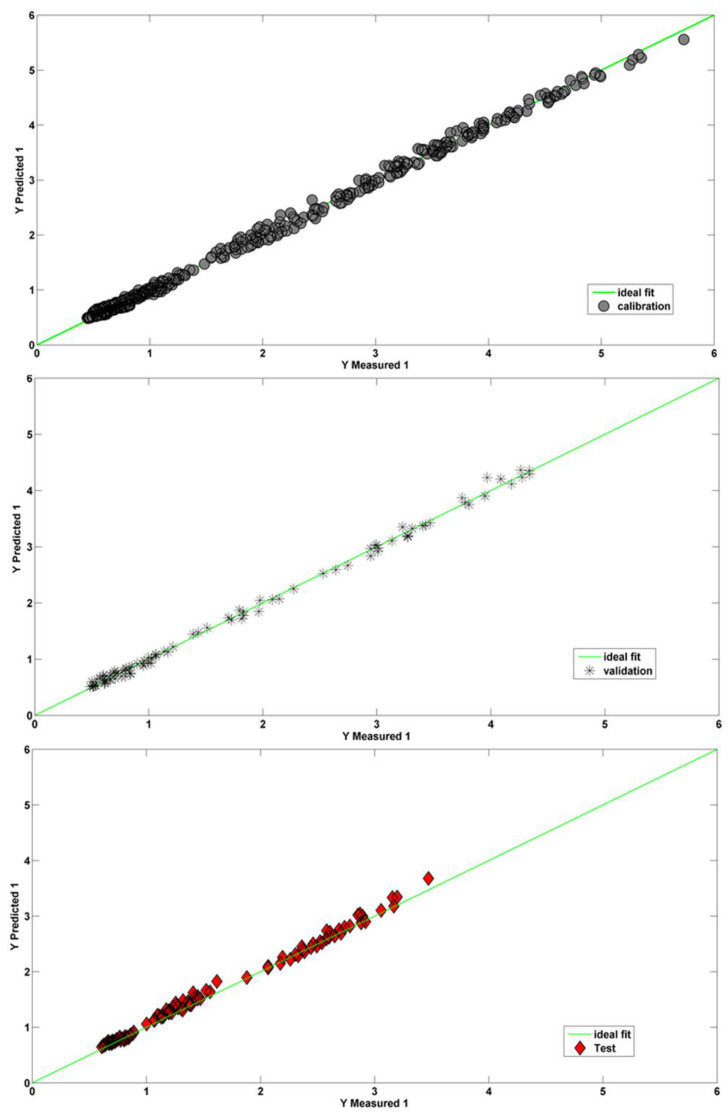
Plot of the predicted *t_r_* obtained by ANN against the experimental values for the calibration, validation, and test sets.

To explore the nonlinear relationship between the retention, the selected molecular descriptors, and the chromatographic parameters, the ANN technique was used to build a retention time predictive model. The networks were generated using the following predictors’ input: MW, nCIR, nR10, RDF090u, RDF030m, and Mor07u (molecular descriptors); and F, T, and t_g_ (chromatographic parameters). The target variable was t_r_ as the output neuron. In the design of the ANN, the dataset was divided into three groups: training, validation, and test sets. A three-layer network with a hyperbolic tangent transfer function for the hidden layer and an identity function for the output layer was used. The network was then instructed using the training set by the backpropagation strategy for optimization of the weights, randomly initialized between −1 and 1, and bias values. A different number of nodes in the hidden layer was tested and the best ANN architecture was composed of nine input neurons, two hidden neurons, and one output neuron. The Root-Mean-Square Error (*RMSE*) value, calculated as in Equation (1), measures the quality of the outputs relative to the target values; it is determined by squaring individual errors, adding them, dividing the sum by their total number, and then calculating the square root of this quantity. Therefore, the RMSE gives a single number that summarizes the overall error of the model, and it was used to measure and compare the accuracy of the predictions in the training, validation, and test sets:

(1) $$RMSE=\sqrt{\frac{1}{n}\overset{n}{\underset{i=1}{\sum }}(t_{r}-{\hat{t}}_{r})}$$

where $t_{r}\;$, ${\hat{t}}_{r},$ and *n* represent the experimental and the calculated value of the retention time and the number of samples, respectively. It should be noted that to evaluate overfitting, the network training should stop when the RMSE of the validation set begins to increase contrary to the *RMSE* of the calibration set, whose value is continuously decreasing. The *RMSE* values for the training, validation, and test sets are 0.074, 0.065, and 0.077, respectively. The optimum architecture was tested 100 times and the results were averaged to ensure that the best model was not caused by a certain initial weight model. The predictive performance of this network was tested on the three external analytes, i.e., fluorene, pyrene, and benzo[b] fluoranthene. The coefficient of determination R^2^ for the calibration, validation and test sets were 0.9972, 0.9973, and 0.9975, respectively. Inspection of the results shows the high prediction performances of this model. The plots of predicted retention times versus the experimental values for the calibration, validation, and test sets are reported in Figure 2.

The graphs show a clear agreement between *t* and ${\hat{t}}_{r}$; moreover, a very low dispersion around the straight line with the unit slope is evident. These considerations can be extended to all three data sets. These results clearly show the feasibility of using ANNs as a regression method for predicting chromatographic retention. This approach makes it possible to construct a nonlinear model in which molecular descriptors are good predictors. 

Radial distribution function descriptors or RDF descriptors can be interpreted as the probability distribution to find an atom in a spherical volume of radius R. The RDF descriptors are based on the distance distribution in the geometrical representation of a molecule and provide information about interatomic distances. The numeric code indicates an interatomic distance, e.g., 030 corresponding to 3.0 Å, which is the probability of finding an interatomic distance of 3.0 Å. RDF descriptors provide information about the distribution of interatomic lengths in the entire molecule, for example, bond distances, ring types, planar and nonplanar systems, and atom types. These molecular characteristics are closely related to the chromatographic retention mechanisms. For this reason, RDF descriptors are particularly valuable in quantitative structure–retention studies. The RDF090u and RDF030m descriptors can provide information about the shape and size of a molecule, which can influence its interaction with the stationary phase in RP-HPLC. For example, a PAH with a larger RDF090u descriptor value may have a more extended shape and may interact more strongly with the stationary phase, resulting in a longer retention time. On the other hand, a PAH with a larger RDF030m descriptor value may have a more compact shape and may interact less strongly with the stationary phase, resulting in a shorter retention time. Mor07u is a 3D-Molecule Representation of Structures based on Electron diffraction (3D-MoRSE) descriptor, which provides information derived from the three-dimensional coordinates; it shows great potential for the representation of molecular structures. A typical MoRSE descriptor is accompanied by a number which refers to the scattering parameter and a letter indicating the type of weighting. Consequently, Mor07u stands for a descriptor with the scattering parameter equal to 7 Å. 3D-MoRSE descriptors are typically evaluated with various weights: weighted with atomic van der Waals volume, atomic mass, atomic Sanderson electronegativity, atomic polarizability, and unweighted 3D-MoRSE. The relationship between 3D-MoRSE descriptors and retention in HPLC is complex and dependent on several factors, including the composition of the mobile and stationary phases, the temperature, and the properties of the compounds being analyzed. In general, compounds with higher 3D-MoRSE values may have stronger interactions with the stationary phase, leading to longer retention times in HPLC. This is because the 3D-MoRSE descriptor is related to the electronic structure of the molecule, which can influence its ability to interact with the stationary phase. For example, molecules with high 3D-MoRSE values may have more polarizable electrons or a larger dipole moment, which can result in stronger interactions with polar stationary phases.

The descriptors nCIR and nR10 are constitutional descriptors and are commonly used because they reflect the molecular composition of a compound. The nR10 descriptor illustrates the presence of independent or 10-member fused rings in molecules. It is particularly useful for the description of condensed aromatic rings and plays an important role in the determination of their physicochemical properties. Eventually, the nCIR descriptor represents the number of the circuit and includes both rings and circuits. These two descriptors help provide useful information for differentiating the structures of the analytes examined. The hydrophobicity of PAHs is generally determined by the size and number of the aromatic rings in their structure. Thus, the nCIR and nR10 descriptors can provide insight into the hydrophobicity of a PAH molecule and its expected retention behavior in RP-HPLC. In general, larger and more complex PAHs with higher numbers of rings are expected to have stronger hydrophobic interactions with the stationary phase, resulting in longer retention times in RP-HPLC. Molecular weight is calculated as the sum of the atomic weights and is an important descriptor related to the size of the molecules; therefore, it is useful for discriminating homologous molecules belonging to the same class. Larger PAHs tend to have more nonpolar surface area and are thus more strongly retained by the stationary phase.

### 2.3. QSRR-PLS Model

QSRR-PLS models were calibrated on a training set consisting of 80% of the samples. Then, the validation of the regression models was performed on an external set, collecting the remaining samples. In order to ensure the representativeness of both data sets, the splitting between training and validation sets was performed using the duplex Kennard–Stone algorithm [57]. The performances of all the regression approaches tested are reported in Table 3 in terms of the Root-Mean-Square Error (RMSECV) and determination coefficient (R^2^_cv_), both calculated by cross-validation on the training set (venetian blinds, five cancellation groups); the RMSE calculated for the prediction of ***Y*** response on the test set (RMSEP) is also reported for the optimal models. 

PLS models were firstly calculated on the whole data matrix, testing two different pretreatments in order to define the most suitable one. The best regression performance was obtained when the autoscaled ***X*** matrix was handled; nevertheless, the results were not sufficiently accurate (RMSECV = 0.252, R^2^_cv_ = 0.967, RMSEP = 0.601).

Eventually, in order to investigate whether feature selection would improve the predictive ability of the models, three different approaches (described in Section 3.4.3), VIP analysis, Covariance Selection, and genetic algorithms, were tested. Consequently, each tool was applied, and the number of predictors reduced in agreement with their outcomes.

**Figure 3:**
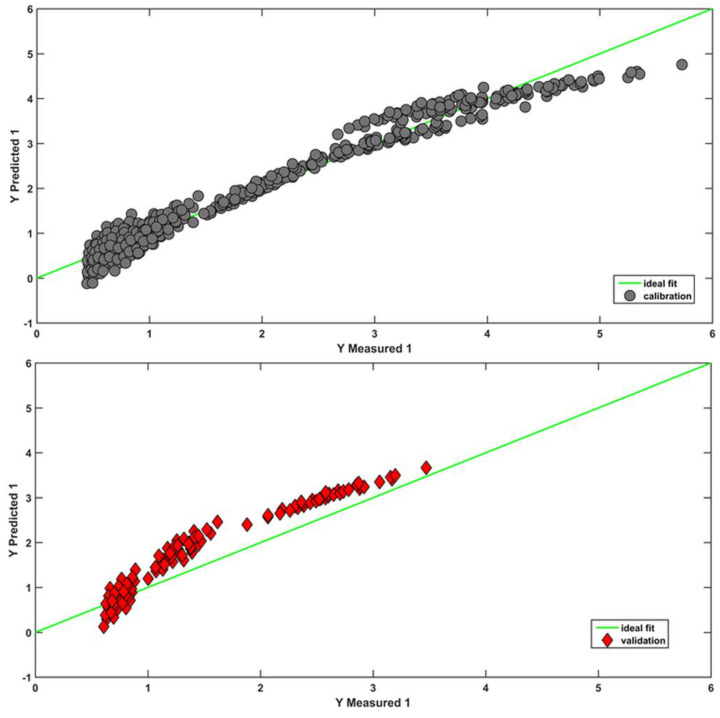
PLS model on the predictors selected by CovSel.

Once the VIP indices were estimated for all the available variables, only those presenting a value higher than one were retained; this led to the selection of 70 molecular descriptors. The regression model calibrated on the (autoscaled) reduced training matrix led to an improvement of the model performances, but still not completely satisfying ones (RMSECV = 0.322, R^2^cv = 0.945, RMSEP = 0.541). Compared to VIP analysis, CovSel is (by its own nature) a much more parsimonious variable selection approach, and, not surprisingly, it pointed out only 11 variables to be retained: the three experimental variables (T, t, and F), the molecular weight (also selected by the GA-ANN strategy), SM09_AEA (dm), SpPosA_RG, RDF110u (RDF090u and RDF030m were selected also by the GA-ANN strategy), Mor20u, Mor26u, Mor30u (Mor07u was selected by the GA-ANN strategy), and H7u, indicating that the most relevant information is associated with the experimental features, molecular weights, edge adjacencies, 3D/3D-MoRSE descriptors, and radial distribution function descriptors. Table S1 (Supplementary Materials) shows the values of these descriptors calculated for all the analytes. Moreover, CovSel led to the best solution using the PLS approach, as shown by an RMSECV value of 0.246 and by the agreement between the measured and predicted ***Y*** responses shown in the Figure 3; the regression coefficients are reported in Table S2. 

Despite this, a comparison of the residuals produced by the different approaches (as can be seen by comparing the two reported figures) revealed that the linear approach is not sufficient to describe the phenomenon. This result is probably linked to the introduction of chromatographic parameters into the model. Indeed, a nonlinear trend was found in the works where chromatographic parameters were included in QSRR studies. The ANN technique, in this case, proved to be more appropriate in predicting the retention times of this class of compounds, as confirmed by the residues that are mainly distributed around zero in Figure 3. Eventually, further PLS models were built on the data set including only the predictors selected by the GA (see Section 2.1). This strategy did not provide particularly accurate predictions, indicating that the most suitable PLS-based solution is the one provided by the application of Covariance Selection.

## 3. Materials and Methods

### 3.1. Chemicals and Reagent

The standard PAHs mixture TraceCERT^®^, 10 μg/mL of each component in acetonitrile (Sigma–Aldrich, St. Louis, MO, USA), was used. The mixture consisted of acenaphthene (Ac), Acenaphthylene (Ap), Anthracene (A), Benz[a]anthracene (BaA), Benzo[a]pyrene (BaP), Benzo[b]fluoranthene (BbF), Benzo[k]fluoranthene (BkF), Benzo[g,h,i]perylene (BghiP), Chrysene (Ch), Dibenz[a,h]anthracene (DBahA), Fluoranthene (Fl), Fluorene (F), Indeno[1,2,3-cd]pyrene (IP), Naphthalene (Na), Phenanthrene (Pa), and Pyrene (P). A standard solution (5 µg/mL of each analyte) was prepared by dilution in acetonitrile HPLC-grade Chromasolv® (Sigma–Aldrich, St. Louis, MO, USA) and stored at 4 °C. The mobile phase was prepared by mixing acetonitrile and ultra-pure water, generated by a Milli-Q System (Millipore, Bedford, MA, USA).

### 3.2. UHPLC-DAD Conditions and Design of Experiments

The UHPLC analysis was carried out using an Acquity H-Class UHPLC system (Waters, Milford, MA, USA) equipped with a degassing system, a quaternary solvent manager, a sample manager, a column heater, and a photodiode array detector set in the range of 220–350 nm. Data processing was managed by Empower v.3.0 software (Waters). The mobile phase consisted of MilliQ water (eluent A), and acetonitrile (eluent B) was dispensed according to the following linear gradient profile: 60% B to 100% B in a variable time *t_g_* from 4 and 8 min; 100% B kept for 1 min; and 100% B to the initial composition in 2 min. The column was re-equilibrated for 2 min before successive analysis. The eluent flow rate (F) was investigated between 0.6 and 0.8 mL/min. An amount of 1 μL of PAH standard solution at 5 μg/μL was injected into the UHPLC system equipped with a reversed-phase column Kinetex C18 (Phenomenex, Torrance, CA, USA) with 100 mm length, 4.6 mm internal diameter, and 2.6 μm particle size, protected by a C18 pre-column SecurityGuard ULTRA (Phenomenex, Torrance, CA, USA). The column oven was maintained at temperatures between 25 and 35 °C and the samples were kept at 15 °C. For the present study, three independent chromatographic variables were selected, which included the eluent flow rate (F), the temperature of the column (T), and the duration of the linear step of the gradient (t_g_). The chromatographic conditions, reported in Table S3, were chosen according to a three-level full-factorial design with eight additional experiments performed in the central points of the eight cubic subspaces.

### 3.3. Computation of Molecular Descriptors

Molecular descriptors can be obtained simply by the addition of given atomic contributions. In the most complex cases, the information contained in the three-dimensional molecular geometry should be developed to extract the structural properties. Moreover, QSAR/QSPR analysis can take advantage of several software packages able to complete the computations of a large number of theoretical molecular descriptors. 

In this work, the starting geometries of the PAHs were generated by the MacroModel 7.1 molecular modelling program package [58]. By using the MM2 forcefield, a conformational search was carried out to identify the global energy minimum for each molecule. Dragon software (version 5.4) [44] was used to compute the molecular descriptors from the optimized geometries. This version provided 4885 descriptors belonging to 29 classes: zero- (0D), one- (1D), two- (2D), and three-dimensional (3D) descriptors, depending on whether they were computed starting from the chemical formula, the substructure list representation, the molecular graph, or the geometrical representation of the molecule [59]. The quantities with little variance were eliminated, and only one descriptor was retained among groups of highly correlated ones (r > 0.95). A total of 550 molecular descriptors belonging to various classes remained after this procedure. 

### 3.4. Multivariate Calibration

#### 3.4.1. Artificial Neural Network

**Figure 4:**
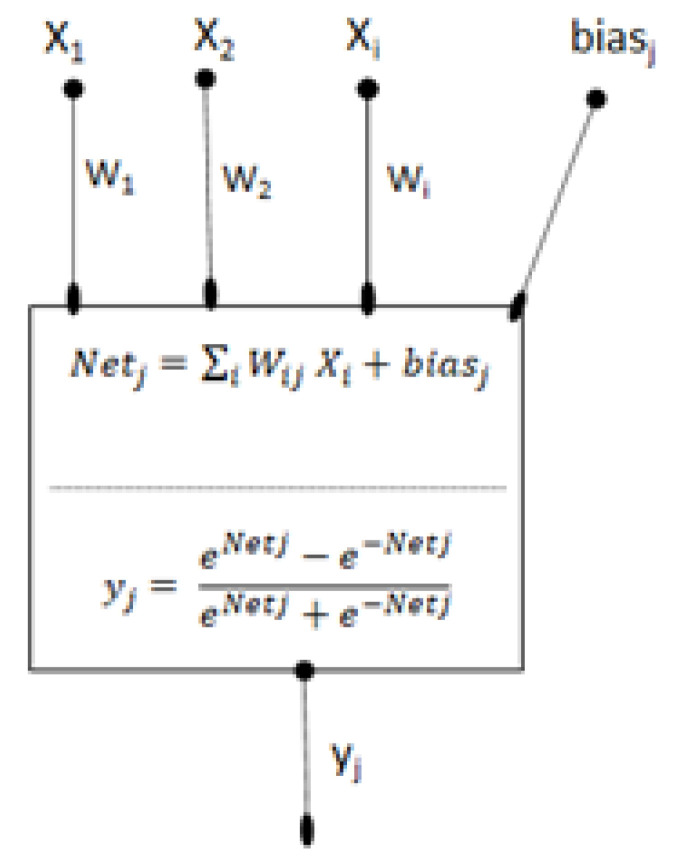
General scheme of a neuron unit. Xi represent the variables of the input layer, *Wij* is the weight from neuron *j* and the neuron *i*, and *bias_j_* is the bias on neuron *j*. *Net_j_* is the activation as the sum of the weighted inputs of neuron *j*. *y_j_* is the output of neuron *j* resulting from the application of hyperbolic tangent transfer function.

An ANN with a layered structure has a biological background. ANNs are mathematical models designed to imitate the way in which the human brain processes information [60]. The fundamental units are named neurons, generally organized into a layered structure, formed by one input layer, one output layer, and at least one hidden layer. Each neuron in any layer is fully connected with the neurons of the succeeding layer by synapses. In this work, a backpropagation neural network (BNN) was used, which had three layers: one input layer which collects the independent variables, one output neuron providing the retention time (*t_r_*) as response, and one hidden layer with an adjustable number of neurons, as reported in Figure 4.

The strength of the synapse from neuron *i* to neuron j is defined by means of a weight. Furthermore, each neuron j, from the hidden layer and the output neuron, is associated with a real value, named bias. The activation of a neuron is defined as the sum of weighted input signals to that neuron, as reported in Equation (2).

(2) $${Net}_{j}=\underset{i}{\sum }W_{ij}X_{i}+bias_{j}$$

where $W_{ij}$ is the weight connection to neuron j in the actual layer from neuron i in the preceding layer, and ${bias}_{j}$ is the bias on neuron *j*. The activation is transformed to the neuron output by means of a transfer function; this type of function can significantly influence the performance of the network. Thus, it is important to select a type of activation function appropriate for the ANN topology. In this study, we used the hyperbolic tangent function, a sigmoid curve that often performs better than the logistic function because of its symmetry; it is zero-centered and its output ranges between −1 and 1. The optimization of weights was obtained by a mechanism of backpropagation. The goal of the training of a network is to change the weights between the layers in a direction that minimizes the error between the output network and the output target. In order to find the best model complexity, the complete data were divided into three data sets. Three molecules were randomly extracted to be used as an external test set (105 records) while we ensured that the observed retentions of the three compounds as a whole covered the studied retention range. The remaining data were randomly divided into a training set of 364 records (80%) and a validation set of 91 records (20%). Overfitting in ANNs refers to the phenomenon where the model becomes too complex and starts to fit the training data too closely, thereby losing the ability to generalize new, unseen data well. This can happen when the network is trained for too many epochs or has too many parameters relative to the size of the training set. When this happens, the model may perform well on the training data, but its performance on the validation data and test data will be poor. To mitigate the risk of overfitting in ANNs, it is common to use a validation set during the training process. The validation set is a subset of the training data that is not used for training but is instead used to evaluate the performance of the model on unseen data. During the training process, the model is evaluated on the validation set at regular intervals to monitor its performance and detect any signs of overfitting. If the model’s performance on the validation set starts to degrade while its performance on the training set continues to improve, this is a sign of overfitting, and the training process can be stopped [27]. The slightest validation error is indeed a good criterion to stop training the network. Once the optimal network has been chosen, a third external set (the test set) can be used to finally demonstrate its predictive ability. 

#### 3.4.2. Partial Least Squares Regression (PLS-R)

Partial Least Squares (PLS) [61] handles linear regression problems by solving Equation (3):

(3) Y=XB+E

where Y is the response(s) matrix (independent variable(s)), X is the experimental data matrix (dependent variables), B is the regression coefficients matrix, and E is the residuals of the model. The PLS-Regression algorithm works by decomposing the ***X*** and ***Y*** matrices in scores (***T*** and U, respectively) and loadings ones (P and Q, respectively, Equations (4) and (5)):

(4) $$X=TP^{T}+D$$

(5) $$Y=UQ^{T}+F$$

and then searching for a linear inner relationship U=BT between the two resulting score matrices [62]. Once the latent variable space is defined and the bilinear model is built, it can be applied to predict the properties of new samples, belonging to an external validation set.

#### 3.4.3. Variable Selection Tools

In the case of PLS-R and ANN regression models, the reduction of the number of variables is not strictly required, but it can be useful to improve the prediction performances, reduce the risk of overfitting, remove the redundant information, and facilitate the results’ interpretation [63]. Many tools are available, depending on the data set under study, for searching for a good subset of variables, and (internal/cross-) validation (in terms of *Q*^2^ value) is a suitable way to properly individuate it. Variable Importance in Projection, Covariance Selection, and genetic algorithm selection procedures were used in this work and are illustrated below.

Variable Importance in Projection (*VIP*) is probably the most widely used model-based variable importance measure. For the *i* variable, VIP is calculated in Equation (6) as:

(6) $$VIP_{i}=\sqrt{\frac{\sum_{a=1}^{A}w_{ia}^{2}*SSY_{a}*I}{SSY_{tot}*A}}$$

where *A* and *I* are the number of the components in the reduced model and the number of original variables, respectively; *SSY_a_* and *SSY_tot_* are the sum of squares (both calculated from the *X* scores matrix and PLS-coefficients vector) of explained variance for the component *a* and for all the dependent variables, respectively. *w_ia_* is the weight of variable *i* on component *a*, explaining both the covariance between the independent and the dependent variable *i* as well as the importance of variable *i* in the model of the independent ones, summed over the model dimension [64]. Customarily, variables presenting a VIP index smaller than one are not considered relevant to the improvement of the model’s predictions. 

Covariance Selection (CovSel) [65] is a feature selection approach able to face the issues provided by having a (relatively) high number of correlated predictors. Applying this methodology, the selection of the variables is operated step by step on the basis of the covariance within each predictor and *Y*. Firstly, the number of variables to be selected is defined a priori, and then X and Y are mean-centered/autoscaled (obtaining $X_{t}$ and $Y_{t}$). Then, the CovSel algorithm iteratively follows the same steps: The covariance between each $X_{ti}$ variable and $Y_{ti}$ is calculated, and the predictor presenting the highest one ($x_{tsel}$) is selected. Then, $X_{ti}$ and $Y_{ti}$ are orthogonalized with respect to $x_{tsel}$ [66]. The procedure restarts, and all the steps are repeated until the fixed number of selected variables is reached.

Genetic algorithms (GAs) are based on the above Darwinian principles of natural selection and evolution [45,46,47,48,67,68,69,70]. They manipulate a population of potential solutions to an optimization (or search) problem. Specifically, they operate on encoded representations of the solutions, equivalent to the chromosomes of individuals in nature. Each solution is associated with a fitness value that reflects how good it is compared to other solutions in the population. The selection policy is ultimately responsible for ensuring the survival of the best-fitted individuals. Manipulation of the “genetic material” is performed through crossover and mutation operators. The initial population of individuals (models) is usually generated randomly. A chromosome, namely, a binary vector in which each position (gene) encodes the presence or absence of a descriptor by 1 or 0, respectively, represents each model. The starting population evolves through mutation and crossover until an optimal or near-optimal model is identified. The chance for a given chromosome to be preserved in the next generation is associated to the predictive performance of the related model, which is quantified by the determination coefficient in leave-one-out cross-validation (R^2^ loo-cv).

## 4. Conclusions

The study provides an application of QSRR methods to predict the retention times for 16 compounds within the PAH contaminant class. UHPLC equipment was used to collect retention times under various chromatographic conditions. A three-level full factorial design was chosen in order to explore the experimental domain in a representative way. A data set of 560 cases and 4885 descriptors was analyzed by different chemometric methods. Until now, studies involving QSRR analysis of PAHs were carried out in gas chromatography or in HPLC in order to predict different molecular properties. To the best of our knowledge, no UHPLC work is reported in the literature; this method allows for the separation and analysis of PAHs with increased resolution, faster analysis time, and higher detection sensitivity compared to conventional HPLC methods. In addition, this study can be expanded in the future by introducing into the data set additional compounds of interest related to the presence of PAHs in the environment, such as the products resulting from their metabolism; in particular, oxy- and hydroxy-PAHs constitute a class of compounds that are frequently studied in biological monitoring campaigns. Current work has confirmed that GA-ANN is a suitable method for predicting the retention time in the UHPLC apparatus of various polycyclic aromatic hydrocarbons from their molecular descriptors. This approach resulted in a nonlinear, sufficiently generalized model (RMSEP = 0.077), which was based on seven selected descriptors. Two RDF descriptors were chosen, as they are closely related to the chromatographic retention mechanisms; they were combined with a 3D-MoRSE descriptor (related to the three-dimensional coordinates), two ring descriptors, and the molecular weight, which is useful to discriminate homologous molecules belonging to the same class. Therefore, although ANN is a method that could be subject to overfitting, it proved to be applicable when well-optimized and validated. The coefficient of determination for the external test calculated for fluorene, pyrene, and benzo[b] fluoranthene was 0.9975. On the contrary, PLS is less prone to overfitting; CovSel-PLS-R was the most parsimonious approach that also showed sufficiently accurate results. Nevertheless, as can be seen from the residues, in this specific case, a linear approach was not completely appropriate to describe the phenomenon. Further studies may be conducted to assess whether the 3D-MoRSE descriptors, radial distribution function, and ring descriptors are also suitable to describe the chromatographic behavior of molecules with a higher structural variability and with different functional groups.

## Figures and Tables

**Table 1:** Parameters used in GA analysis.

GA Parameter	Selected Option
Initial population size	100 chromosomes
Regression method	Multilinear regression
Response to maximize	Cross-validated % explained variance
Maximum number of descriptors selected in the same chromosome	5–7
Probability of mutation (%)	0.1
Elitism (%)	2
Number of GA runs	50
Stop condition	Maximum number of cycles in each GA run = 10 Maximum number of cycles without response improvement = 5

**Table 2:** Selected descriptors.

Molecular Descriptor	Meaning
MW	Molecular weight
Mor07u	3D-MoRSE descriptor/unweighted
RDF030m	Radial distribution function-030/weighted by mass
RDF 090u	Radial distribution function-090/unweighted
nR10	Ring descriptors
nCIR	Ring descriptors

**Table 3:** Results of Partial Least Squares approach.

Model	Preprocessing	RMSECV	R2cv	RMSEP
PLS	Mean-centering	0.303	0.952	0.435
PLS	Autoscaling	0.252	0.967	0.601
PLS + VIP	Autoscaling	0.322	0.945	0.541
PLS + CovSel	Autoscaling	0.246	0.968	0.433
PLS + GA	Autoscaling	0.271	0.961	0.362

## Data Availability

Data of molecular descriptors are reported in the Supplementary Materials.

## Supplementary Materials

The following supporting information can be downloaded at: https://www.mdpi.com/article/10.3390/molecules28073218/s1, Table S1: Molecular descriptors for PLS model; Table S2: Regression coefficients for PLS model; Table S3: Dataset for ANN model.

## Sample Availability

Samples of the compounds are not available from the authors.
