# Supplementary material for: Quantitative Structure–Retention Relationship Analysis of Polycyclic Aromatic Compounds in Ultra-High Performance Chromatography
Source: Molecules. 2023 Apr 4;28(7):3218. doi: 10.3390/molecules28073218 (PMC10096086; doi:10.3390/molecules28073218)
Supplement: Supplementary file 1 [file molecules-28-03218-s001.zip › molecules-2291506-supplementary.pdf]

Table S1: Molecular descriptors for PLS model

Analite	MW (u.m.a.)	SM09_AEA(dm)	SpPosA_RG	RDF0110u	Mor20u	Mor26u	Mor30u	H7u
Naftalene	128,18	0	0,429	0	1,033	0,785	0,21	0
Acenaphthylene	154,22	0	0,45	0	0,887	0,146	-0,104	0
Acenaphthene	152,2	0	0,453	0	0,883	0,354	-0,234	0
Fluorene	166,23	-2	0,45	0	0,807	0,17	-0,469	0
Phenanthrene	178,24	-2	0,452	0	1,245	0,584	-0,479	0,078
Anthracene	178,24	-2	0,448	0	0,923	0,262	-0,326	0,003
Fluoranthene	202,26	-1,874	0,446	0	1,215	0,028	-0,55	0,036
Pyrene	202,26	-1,717	0,445	0	0,896	0,238	-0,344	0,012
Benzo [a] anthracene	228,3	-1,234	0,447	0,009	1,348	0,324	-0,462	0,174
Chrysene	228,3	-1,386	0,449	0,004	1,412	0,514	-0,307	0,068
Benzo [b] fluoranthene	252,32	-0,95	0,444	0,947	1,426	0,186	-0,662	0,099
Benzo [k] fluoranthene	252,32	-0,894	0,442	1,821	1,594	0,165	-0,636	0,127
Benzo [a]pyrene	252,32	-0,903	0,444	0	1,318	0,41	-0,38	0,037
Dibenzo [a,h]anthracene	278,36	-0,781	0,447	3,154	1,005	0,013	-0,275	0,236
Benzo[g,h,i] perylene	276,34	-0,618	0,44	0	0,997	0,429	-0,358	0,233
Indeno[1,2,3-cd]pyrene	276,34	-0,635	0,44	0,597	1,196	-0,008	-0,72	0,043

Table S2: Regression coefficients for PLS model

molecular descriptor	***CovSel-PLS Coefficients***
MW	1,468
SM09_AEA(dm)	0,212
t	0,188
F	-0,153
T	-0,126
SpPosA_RG	-0,148
Mor20u	-0,299
H7u	-0,236
Mor26u	0,295
RDF110u	0,196
Mor30u	-0,246

Table S3: Dataset for ANN model and subdivision in training set(train), validation set (valid) and test set (test)

set	Analite	T (°C)	t (min)	F (mL/min)	tr (min)	MW (u.m.a.)	nCIR	nR10	RDF090u	RDF030m	Mor07u
valid	Naftalene	25	4	0,6	0,618	128,18	3	1	0	0	3,865
train	Naftalene	25	6	0,6	0,622	128,18	3	1	0	0	3,865
train	Naftalene	25	8	0,6	0,62	128,18	3	1	0	0	3,865
valid	Naftalene	25	4	0,7	0,531	128,18	3	1	0	0	3,865
train	Naftalene	25	6	0,7	0,534	128,18	3	1	0	0	3,865
valid	Naftalene	25	8	0,7	0,534	128,18	3	1	0	0	3,865
train	Naftalene	25	4	0,8	0,464	128,18	3	1	0	0	3,865

train	Naftalene	25	6	0,8	0,468	128,18	3	1	0	0	3,865
train	Naftalene	25	8	0,8	0,471	128,18	3	1	0	0	3,865
valid	Naftalene	30	4	0,6	0,61	128,18	3	1	0	0	3,865
train	Naftalene	30	6	0,6	0,611	128,18	3	1	0	0	3,865
train	Naftalene	30	8	0,6	0,613	128,18	3	1	0	0	3,865
valid	Naftalene	30	4	0,7	0,522	128,18	3	1	0	0	3,865
train	Naftalene	30	6	0,7	0,524	128,18	3	1	0	0	3,865
train	Naftalene	30	8	0,7	0,524	128,18	3	1	0	0	3,865
train	Naftalene	30	4	0,8	0,458	128,18	3	1	0	0	3,865
train	Naftalene	30	6	0,8	0,46	128,18	3	1	0	0	3,865
train	Naftalene	30	8	0,8	0,461	128,18	3	1	0	0	3,865
train	Naftalene	35	4	0,6	0,593	128,18	3	1	0	0	3,865
train	Naftalene	35	6	0,6	0,597	128,18	3	1	0	0	3,865
train	Naftalene	35	8	0,6	0,596	128,18	3	1	0	0	3,865
valid	Naftalene	35	4	0,7	0,51	128,18	3	1	0	0	3,865
valid	Naftalene	35	6	0,7	0,512	128,18	3	1	0	0	3,865
valid	Naftalene	35	8	0,7	0,513	128,18	3	1	0	0	3,865
train	Naftalene	35	4	0,8	0,446	128,18	3	1	0	0	3,865
train	Naftalene	35	6	0,8	0,449	128,18	3	1	0	0	3,865
train	Naftalene	35	8	0,8	0,451	128,18	3	1	0	0	3,865
train	Naftalene	27,5	5	0,65	0,57	128,18	3	1	0	0	3,865
train	Naftalene	27,5	7	0,65	0,573	128,18	3	1	0	0	3,865
train	Naftalene	27,5	5	0,75	0,493	128,18	3	1	0	0	3,865
train	Naftalene	27,5	7	0,75	0,496	128,18	3	1	0	0	3,865
train	Naftalene	32,5	5	0,65	0,56	128,18	3	1	0	0	3,865
train	Naftalene	32,5	7	0,65	0,562	128,18	3	1	0	0	3,865
valid	Naftalene	32,5	5	0,75	0,486	128,18	3	1	0	0	3,865
train	Naftalene	32,5	7	0,75	0,488	128,18	3	1	0	0	3,865
train	Acenaphthylene	25	4	0,6	0,69	154,22	7	1	0	0,837	2,409
train	Acenaphthylene	25	6	0,6	0,7	154,22	7	1	0	0,837	2,409
valid	Acenaphthylene	25	8	0,6	0,7	154,22	7	1	0	0,837	2,409
valid	Acenaphthylene	25	4	0,7	0,596	154,22	7	1	0	0,837	2,409
train	Acenaphthylene	25	6	0,7	0,602	154,22	7	1	0	0,837	2,409
valid	Acenaphthylene	25	8	0,7	0,603	154,22	7	1	0	0,837	2,409
train	Acenaphthylene	25	4	0,8	0,52	154,22	7	1	0	0,837	2,409
train	Acenaphthylene	25	6	0,8	0,527	154,22	7	1	0	0,837	2,409
train	Acenaphthylene	25	8	0,8	0,531	154,22	7	1	0	0,837	2,409
train	Acenaphthylene	30	4	0,6	0,681	154,22	7	1	0	0,837	2,409
train	Acenaphthylene	30	6	0,6	0,684	154,22	7	1	0	0,837	2,409
valid	Acenaphthylene	30	8	0,6	0,687	154,22	7	1	0	0,837	2,409
train	Acenaphthylene	30	4	0,7	0,584	154,22	7	1	0	0,837	2,409
train	Acenaphthylene	30	6	0,7	0,586	154,22	7	1	0	0,837	2,409
train	Acenaphthylene	30	8	0,7	0,587	154,22	7	1	0	0,837	2,409
train	Acenaphthylene	30	4	0,8	0,51	154,22	7	1	0	0,837	2,409
train	Acenaphthylene	30	6	0,8	0,515	154,22	7	1	0	0,837	2,409
train	Acenaphthylene	30	8	0,8	0,517	154,22	7	1	0	0,837	2,409
train	Acenaphthylene	35	4	0,6	0,658	154,22	7	1	0	0,837	2,409
valid	Acenaphthylene	35	6	0,6	0,665	154,22	7	1	0	0,837	2,409
train	Acenaphthylene	35	8	0,6	0,666	154,22	7	1	0	0,837	2,409

train	Acenaphthylene	35	4	0,7	0,566	154,22	7	1	0	0,837	2,409
train	Acenaphthylene	35	6	0,7	0,571	154,22	7	1	0	0,837	2,409
valid	Acenaphthylene	35	8	0,7	0,572	154,22	7	1	0	0,837	2,409
train	Acenaphthylene	35	4	0,8	0,495	154,22	7	1	0	0,837	2,409
valid	Acenaphthylene	35	6	0,8	0,501	154,22	7	1	0	0,837	2,409
train	Acenaphthylene	35	8	0,8	0,503	154,22	7	1	0	0,837	2,409
train	Acenaphthylene	27,5	5	0,65	0,64	154,22	7	1	0	0,837	2,409
train	Acenaphthylene	27,5	7	0,65	0,644	154,22	7	1	0	0,837	2,409
train	Acenaphthylene	27,5	5	0,75	0,553	154,22	7	1	0	0,837	2,409
train	Acenaphthylene	27,5	7	0,75	0,557	154,22	7	1	0	0,837	2,409
train	Acenaphthylene	32,5	5	0,65	0,625	154,22	7	1	0	0,837	2,409
valid	Acenaphthylene	32,5	7	0,65	0,628	154,22	7	1	0	0,837	2,409
train	Acenaphthylene	32,5	5	0,75	0,542	154,22	7	1	0	0,837	2,409
valid	Acenaphthylene	32,5	7	0,75	0,545	154,22	7	1	0	0,837	2,409
train	Acenaphthene	25	4	0,6	0,816	152,2	7	1	0	0,854	2,745
valid	Acenaphthene	25	6	0,6	0,836	152,2	7	1	0	0,854	2,745
valid	Acenaphthene	25	8	0,6	0,841	152,2	7	1	0	0,854	2,745
train	Acenaphthene	25	4	0,7	0,708	152,2	7	1	0	0,854	2,745
train	Acenaphthene	25	6	0,7	0,72	152,2	7	1	0	0,854	2,745
train	Acenaphthene	25	8	0,7	0,725	152,2	7	1	0	0,854	2,745
valid	Acenaphthene	25	4	0,8	0,618	152,2	7	1	0	0,854	2,745
train	Acenaphthene	25	6	0,8	0,631	152,2	7	1	0	0,854	2,745
train	Acenaphthene	25	8	0,8	0,629	152,2	7	1	0	0,854	2,745
train	Acenaphthene	30	4	0,6	0,806	152,2	7	1	0	0,854	2,745
train	Acenaphthene	30	6	0,6	0,815	152,2	7	1	0	0,854	2,745
train	Acenaphthene	30	8	0,6	0,822	152,2	7	1	0	0,854	2,745
train	Acenaphthene	30	4	0,7	0,69	152,2	7	1	0	0,854	2,745
train	Acenaphthene	30	6	0,7	0,699	152,2	7	1	0	0,854	2,745
valid	Acenaphthene	30	8	0,7	0,704	152,2	7	1	0	0,854	2,745
train	Acenaphthene	30	4	0,8	0,606	152,2	7	1	0	0,854	2,745
valid	Acenaphthene	30	6	0,8	0,615	152,2	7	1	0	0,854	2,745
train	Acenaphthene	30	8	0,8	0,62	152,2	7	1	0	0,854	2,745
train	Acenaphthene	35	4	0,6	0,773	152,2	7	1	0	0,854	2,745
train	Acenaphthene	35	6	0,6	0,79	152,2	7	1	0	0,854	2,745
valid	Acenaphthene	35	8	0,6	0,795	152,2	7	1	0	0,854	2,745
train	Acenaphthene	35	4	0,7	0,669	152,2	7	1	0	0,854	2,745
train	Acenaphthene	35	6	0,7	0,679	152,2	7	1	0	0,854	2,745
train	Acenaphthene	35	8	0,7	0,684	152,2	7	1	0	0,854	2,745
train	Acenaphthene	35	4	0,8	0,589	152,2	7	1	0	0,854	2,745
train	Acenaphthene	35	6	0,8	0,597	152,2	7	1	0	0,854	2,745
train	Acenaphthene	35	8	0,8	0,6	152,2	7	1	0	0,854	2,745
valid	Acenaphthene	27,5	5	0,65	0,761	152,2	7	1	0	0,854	2,745
train	Acenaphthene	27,5	7	0,65	0,772	152,2	7	1	0	0,854	2,745
valid	Acenaphthene	27,5	5	0,75	0,659	152,2	7	1	0	0,854	2,745
valid	Acenaphthene	27,5	7	0,75	0,667	152,2	7	1	0	0,854	2,745
train	Acenaphthene	32,5	5	0,65	0,742	152,2	7	1	0	0,854	2,745
train	Acenaphthene	32,5	7	0,65	0,75	152,2	7	1	0	0,854	2,745
train	Acenaphthene	32,5	5	0,75	0,645	152,2	7	1	0	0,854	2,745
train	Acenaphthene	32,5	7	0,75	0,652	152,2	7	1	0	0,854	2,745

test	Fluorene	25	4	0,6	0,857	166,23	6	0	0,9	0,709	2,938
test	Fluorene	25	6	0,6	0,881	166,23	6	0	0,9	0,709	2,938
test	Fluorene	25	8	0,6	0,888	166,23	6	0	0,9	0,709	2,938
test	Fluorene	25	4	0,7	0,744	166,23	6	0	0,9	0,709	2,938
test	Fluorene	25	6	0,7	0,76	166,23	6	0	0,9	0,709	2,938
test	Fluorene	25	8	0,7	0,766	166,23	6	0	0,9	0,709	2,938
test	Fluorene	25	4	0,8	0,648	166,23	6	0	0,9	0,709	2,938
test	Fluorene	25	6	0,8	0,666	166,23	6	0	0,9	0,709	2,938
test	Fluorene	25	8	0,8	0,664	166,23	6	0	0,9	0,709	2,938
test	Fluorene	30	4	0,6	0,842	166,23	6	0	0,9	0,709	2,938
test	Fluorene	30	6	0,6	0,855	166,23	6	0	0,9	0,709	2,938
test	Fluorene	30	8	0,6	0,862	166,23	6	0	0,9	0,709	2,938
test	Fluorene	30	4	0,7	0,724	166,23	6	0	0,9	0,709	2,938
test	Fluorene	30	6	0,7	0,733	166,23	6	0	0,9	0,709	2,938
test	Fluorene	30	8	0,7	0,739	166,23	6	0	0,9	0,709	2,938
test	Fluorene	30	4	0,8	0,633	166,23	6	0	0,9	0,709	2,938
test	Fluorene	30	6	0,8	0,645	166,23	6	0	0,9	0,709	2,938
test	Fluorene	30	8	0,8	0,651	166,23	6	0	0,9	0,709	2,938
test	Fluorene	35	4	0,6	0,805	166,23	6	0	0,9	0,709	2,938
test	Fluorene	35	6	0,6	0,822	166,23	6	0	0,9	0,709	2,938
test	Fluorene	35	8	0,6	0,827	166,23	6	0	0,9	0,709	2,938
test	Fluorene	35	4	0,7	0,695	166,23	6	0	0,9	0,709	2,938
test	Fluorene	35	6	0,7	0,707	166,23	6	0	0,9	0,709	2,938
test	Fluorene	35	8	0,7	0,712	166,23	6	0	0,9	0,709	2,938
test	Fluorene	35	4	0,8	0,608	166,23	6	0	0,9	0,709	2,938
test	Fluorene	35	6	0,8	0,622	166,23	6	0	0,9	0,709	2,938
test	Fluorene	35	8	0,8	0,627	166,23	6	0	0,9	0,709	2,938
test	Fluorene	27,5	5	0,65	0,8	166,23	6	0	0,9	0,709	2,938
test	Fluorene	27,5	7	0,65	0,812	166,23	6	0	0,9	0,709	2,938
test	Fluorene	27,5	5	0,75	0,692	166,23	6	0	0,9	0,709	2,938
test	Fluorene	27,5	7	0,75	0,702	166,23	6	0	0,9	0,709	2,938
test	Fluorene	32,5	5	0,65	0,774	166,23	6	0	0,9	0,709	2,938
test	Fluorene	32,5	7	0,65	0,784	166,23	6	0	0,9	0,709	2,938
test	Fluorene	32,5	5	0,75	0,673	166,23	6	0	0,9	0,709	2,938
test	Fluorene	32,5	7	0,75	0,682	166,23	6	0	0,9	0,709	2,938
train	Phenanthrene	25	4	0,6	0,988	178,24	6	2	5	1,133	2,216
train	Phenanthrene	25	6	0,6	1,026	178,24	6	2	5	1,133	2,216
train	Phenanthrene	25	8	0,6	1,04	178,24	6	2	5	1,133	2,216
train	Phenanthrene	25	4	0,7	0,861	178,24	6	2	5	1,133	2,216
train	Phenanthrene	25	6	0,7	0,889	178,24	6	2	5	1,133	2,216
train	Phenanthrene	25	8	0,7	0,9	178,24	6	2	5	1,133	2,216
train	Phenanthrene	25	4	0,8	0,75	178,24	6	2	5	1,133	2,216
train	Phenanthrene	25	6	0,8	0,778	178,24	6	2	5	1,133	2,216
train	Phenanthrene	25	8	0,8	0,778	178,24	6	2	5	1,133	2,216
train	Phenanthrene	30	4	0,6	0,964	178,24	6	2	5	1,133	2,216
train	Phenanthrene	30	6	0,6	0,987	178,24	6	2	5	1,133	2,216
train	Phenanthrene	30	8	0,6	1,001	178,24	6	2	5	1,133	2,216
valid	Phenanthrene	30	4	0,7	0,83	178,24	6	2	5	1,133	2,216
valid	Phenanthrene	30	6	0,7	0,848	178,24	6	2	5	1,133	2,216

train	Phenanthrene	30	8	0,7	0,859	178,24	6	2	5	1,133	2,216
valid	Phenanthrene	30	4	0,8	0,728	178,24	6	2	5	1,133	2,216
train	Phenanthrene	30	6	0,8	0,747	178,24	6	2	5	1,133	2,216
train	Phenanthrene	30	8	0,8	0,759	178,24	6	2	5	1,133	2,216
train	Phenanthrene	35	4	0,6	0,914	178,24	6	2	5	1,133	2,216
train	Phenanthrene	35	6	0,6	0,941	178,24	6	2	5	1,133	2,216
train	Phenanthrene	35	8	0,6	0,95	178,24	6	2	5	1,133	2,216
valid	Phenanthrene	35	4	0,7	0,79	178,24	6	2	5	1,133	2,216
train	Phenanthrene	35	6	0,7	0,81	178,24	6	2	5	1,133	2,216
train	Phenanthrene	35	8	0,7	0,82	178,24	6	2	5	1,133	2,216
train	Phenanthrene	35	4	0,8	0,693	178,24	6	2	5	1,133	2,216
valid	Phenanthrene	35	6	0,8	0,715	178,24	6	2	5	1,133	2,216
train	Phenanthrene	35	8	0,8	0,724	178,24	6	2	5	1,133	2,216
train	Phenanthrene	27,5	5	0,65	0,926	178,24	6	2	5	1,133	2,216
train	Phenanthrene	27,5	7	0,65	0,947	178,24	6	2	5	1,133	2,216
valid	Phenanthrene	27,5	5	0,75	0,802	178,24	6	2	5	1,133	2,216
train	Phenanthrene	27,5	7	0,75	0,819	178,24	6	2	5	1,133	2,216
train	Phenanthrene	32,5	5	0,65	0,888	178,24	6	2	5	1,133	2,216
train	Phenanthrene	32,5	7	0,65	0,905	178,24	6	2	5	1,133	2,216
valid	Phenanthrene	32,5	5	0,75	0,774	178,24	6	2	5	1,133	2,216
train	Phenanthrene	32,5	7	0,75	0,789	178,24	6	2	5	1,133	2,216
train	Anthracene	25	4	0,6	1,139	178,24	6	2	0,2	0,662	2,312
train	Anthracene	25	6	0,6	1,197	178,24	6	2	0,2	0,662	2,312
train	Anthracene	25	8	0,6	1,222	178,24	6	2	0,2	0,662	2,312
valid	Anthracene	25	4	0,7	0,998	178,24	6	2	0,2	0,662	2,312
train	Anthracene	25	6	0,7	1,042	178,24	6	2	0,2	0,662	2,312
valid	Anthracene	25	8	0,7	1,061	178,24	6	2	0,2	0,662	2,312
train	Anthracene	25	4	0,8	0,866	178,24	6	2	0,2	0,662	2,312
train	Anthracene	25	6	0,8	0,911	178,24	6	2	0,2	0,662	2,312
train	Anthracene	25	8	0,8	0,94	178,24	6	2	0,2	0,662	2,312
train	Anthracene	30	4	0,6	1,102	178,24	6	2	0,2	0,662	2,312
train	Anthracene	30	6	0,6	1,14	178,24	6	2	0,2	0,662	2,312
train	Anthracene	30	8	0,6	1,162	178,24	6	2	0,2	0,662	2,312
valid	Anthracene	30	4	0,7	0,953	178,24	6	2	0,2	0,662	2,312
train	Anthracene	30	6	0,7	0,981	178,24	6	2	0,2	0,662	2,312
train	Anthracene	30	8	0,7	1,001	178,24	6	2	0,2	0,662	2,312
train	Anthracene	30	4	0,8	0,836	178,24	6	2	0,2	0,662	2,312
train	Anthracene	30	6	0,8	0,867	178,24	6	2	0,2	0,662	2,312
train	Anthracene	30	8	0,8	0,886	178,24	6	2	0,2	0,662	2,312
train	Anthracene	35	4	0,6	1,034	178,24	6	2	0,2	0,662	2,312
train	Anthracene	35	6	0,6	1,075	178,24	6	2	0,2	0,662	2,312
train	Anthracene	35	8	0,6	1,09	178,24	6	2	0,2	0,662	2,312
train	Anthracene	35	4	0,7	0,896	178,24	6	2	0,2	0,662	2,312
train	Anthracene	35	6	0,7	0,928	178,24	6	2	0,2	0,662	2,312
valid	Anthracene	35	8	0,7	0,944	178,24	6	2	0,2	0,662	2,312
train	Anthracene	35	4	0,8	0,788	178,24	6	2	0,2	0,662	2,312
train	Anthracene	35	6	0,8	0,821	178,24	6	2	0,2	0,662	2,312
train	Anthracene	35	8	0,8	0,835	178,24	6	2	0,2	0,662	2,312
train	Anthracene	27,5	5	0,65	1,072	178,24	6	2	0,2	0,662	2,312

train	Anthracene	27,5	7	0,65	1,105	178,24	6	2	0,2	0,662	2,312
train	Anthracene	27,5	5	0,75	0,93	178,24	6	2	0,2	0,662	2,312
train	Anthracene	27,5	7	0,75	0,957	178,24	6	2	0,2	0,662	2,312
valid	Anthracene	32,5	5	0,65	1,018	178,24	6	2	0,2	0,662	2,312
train	Anthracene	32,5	7	0,65	1,044	178,24	6	2	0,2	0,662	2,312
train	Anthracene	32,5	5	0,75	0,889	178,24	6	2	0,2	0,662	2,312
train	Anthracene	32,5	7	0,75	0,913	178,24	6	2	0,2	0,662	2,312
train	Fluoranthene	25	4	0,6	1,302	202,26	12	1	1,8	0,868	4
train	Fluoranthene	25	6	0,6	1,387	202,26	12	1	1,8	0,868	4
valid	Fluoranthene	25	8	0,6	1,433	202,26	12	1	1,8	0,868	4
train	Fluoranthene	25	4	0,7	1,146	202,26	12	1	1,8	0,868	4
valid	Fluoranthene	25	6	0,7	1,213	202,26	12	1	1,8	0,868	4
train	Fluoranthene	25	8	0,7	1,245	202,26	12	1	1,8	0,868	4
valid	Fluoranthene	25	4	0,8	0,996	202,26	12	1	1,8	0,868	4
valid	Fluoranthene	25	6	0,8	1,063	202,26	12	1	1,8	0,868	4
train	Fluoranthene	25	8	0,8	1,108	202,26	12	1	1,8	0,868	4
train	Fluoranthene	30	4	0,6	1,255	202,26	12	1	1,8	0,868	4
train	Fluoranthene	30	6	0,6	1,314	202,26	12	1	1,8	0,868	4
train	Fluoranthene	30	8	0,6	1,351	202,26	12	1	1,8	0,868	4
train	Fluoranthene	30	4	0,7	1,095	202,26	12	1	1,8	0,868	4
valid	Fluoranthene	30	6	0,7	1,135	202,26	12	1	1,8	0,868	4
train	Fluoranthene	30	8	0,7	1,167	202,26	12	1	1,8	0,868	4
valid	Fluoranthene	30	4	0,8	0,96	202,26	12	1	1,8	0,868	4
train	Fluoranthene	30	6	0,8	1,008	202,26	12	1	1,8	0,868	4
train	Fluoranthene	30	8	0,8	1,037	202,26	12	1	1,8	0,868	4
train	Fluoranthene	35	4	0,6	1,171	202,26	12	1	1,8	0,868	4
train	Fluoranthene	35	6	0,6	1,232	202,26	12	1	1,8	0,868	4
train	Fluoranthene	35	8	0,6	1,26	202,26	12	1	1,8	0,868	4
train	Fluoranthene	35	4	0,7	1,019	202,26	12	1	1,8	0,868	4
train	Fluoranthene	35	6	0,7	1,068	202,26	12	1	1,8	0,868	4
train	Fluoranthene	35	8	0,7	1,094	202,26	12	1	1,8	0,868	4
valid	Fluoranthene	35	4	0,8	0,899	202,26	12	1	1,8	0,868	4
train	Fluoranthene	35	6	0,8	0,949	202,26	12	1	1,8	0,868	4
train	Fluoranthene	35	8	0,8	0,971	202,26	12	1	1,8	0,868	4
train	Fluoranthene	27,5	5	0,65	1,235	202,26	12	1	1,8	0,868	4
train	Fluoranthene	27,5	7	0,65	1,287	202,26	12	1	1,8	0,868	4
train	Fluoranthene	27,5	5	0,75	1,076	202,26	12	1	1,8	0,868	4
train	Fluoranthene	27,5	7	0,75	1,118	202,26	12	1	1,8	0,868	4
valid	Fluoranthene	32,5	5	0,65	1,167	202,26	12	1	1,8	0,868	4
train	Fluoranthene	32,5	7	0,65	1,209	202,26	12	1	1,8	0,868	4
valid	Fluoranthene	32,5	5	0,75	1,024	202,26	12	1	1,8	0,868	4
valid	Fluoranthene	32,5	7	0,75	1,061	202,26	12	1	1,8	0,868	4
test	Pyrene	25	4	0,6	1,447	202,26	14	5	0	1,106	2,029
test	Pyrene	25	6	0,6	1,557	202,26	14	5	0	1,106	2,029
test	Pyrene	25	8	0,6	1,618	202,26	14	5	0	1,106	2,029
test	Pyrene	25	4	0,7	1,278	202,26	14	5	0	1,106	2,029
test	Pyrene	25	6	0,7	1,365	202,26	14	5	0	1,106	2,029
test	Pyrene	25	8	0,7	1,409	202,26	14	5	0	1,106	2,029
test	Pyrene	25	4	0,8	1,113	202,26	14	5	0	1,106	2,029

test	Pyrene	25	6	0,8	1,199	202,26	14	5	0	1,106	2,029
test	Pyrene	25	8	0,8	1,257	202,26	14	5	0	1,106	2,029
test	Pyrene	30	4	0,6	1,392	202,26	14	5	0	1,106	2,029
test	Pyrene	30	6	0,6	1,473	202,26	14	5	0	1,106	2,029
test	Pyrene	30	8	0,6	1,524	202,26	14	5	0	1,106	2,029
test	Pyrene	30	4	0,7	1,22	202,26	14	5	0	1,106	2,029
test	Pyrene	30	6	0,7	1,276	202,26	14	5	0	1,106	2,029
test	Pyrene	30	8	0,7	1,32	202,26	14	5	0	1,106	2,029
test	Pyrene	30	4	0,8	1,072	202,26	14	5	0	1,106	2,029
test	Pyrene	30	6	0,8	1,135	202,26	14	5	0	1,106	2,029
test	Pyrene	30	8	0,8	1,174	202,26	14	5	0	1,106	2,029
test	Pyrene	35	4	0,6	1,318	202,26	14	5	0	1,106	2,029
test	Pyrene	35	6	0,6	1,379	202,26	14	5	0	1,106	2,029
test	Pyrene	35	8	0,6	1,417	202,26	14	5	0	1,106	2,029
test	Pyrene	35	4	0,7	1,132	202,26	14	5	0	1,106	2,029
test	Pyrene	35	6	0,7	1,197	202,26	14	5	0	1,106	2,029
test	Pyrene	35	8	0,7	1,234	202,26	14	5	0	1,106	2,029
test	Pyrene	35	4	0,8	1,002	202,26	14	5	0	1,106	2,029
test	Pyrene	35	6	0,8	1,066	202,26	14	5	0	1,106	2,029
test	Pyrene	35	8	0,8	1,097	202,26	14	5	0	1,106	2,029
test	Pyrene	27,5	5	0,65	1,381	202,26	14	5	0	1,106	2,029
test	Pyrene	27,5	7	0,65	1,45	202,26	14	5	0	1,106	2,029
test	Pyrene	27,5	5	0,75	1,207	202,26	14	5	0	1,106	2,029
test	Pyrene	27,5	7	0,75	1,263	202,26	14	5	0	1,106	2,029
test	Pyrene	32,5	5	0,65	1,304	202,26	14	5	0	1,106	2,029
test	Pyrene	32,5	7	0,65	1,36	202,26	14	5	0	1,106	2,029
test	Pyrene	32,5	5	0,75	1,147	202,26	14	5	0	1,106	2,029
test	Pyrene	32,5	7	0,75	1,196	202,26	14	5	0	1,106	2,029
train	Benzo [a] anthracene	25	4	0,6	2,013	228,3	10	3	2,9	1,911	3,029
train	Benzo [a] anthracene	25	6	0,6	2,271	228,3	10	3	2,9	1,911	3,029
train	Benzo [a] anthracene	25	8	0,6	2,437	228,3	10	3	2,9	1,911	3,029
train	Benzo [a] anthracene	25	4	0,7	1,811	228,3	10	3	2,9	1,911	3,029
train	Benzo [a] anthracene	25	6	0,7	2,029	228,3	10	3	2,9	1,911	3,029
train	Benzo [a] anthracene	25	8	0,7	2,157	228,3	10	3	2,9	1,911	3,029
train	Benzo [a] anthracene	25	4	0,8	1,586	228,3	10	3	2,9	1,911	3,029
valid	Benzo [a] anthracene	25	6	0,8	1,795	228,3	10	3	2,9	1,911	3,029
train	Benzo [a] anthracene	25	8	0,8	1,953	228,3	10	3	2,9	1,911	3,029
train	Benzo [a] anthracene	30	4	0,6	1,911	228,3	10	3	2,9	1,911	3,029
train	Benzo [a] anthracene	30	6	0,6	2,11	228,3	10	3	2,9	1,911	3,029
train	Benzo [a] anthracene	30	8	0,6	2,245	228,3	10	3	2,9	1,911	3,029
valid	Benzo [a] anthracene	30	4	0,7	1,7	228,3	10	3	2,9	1,911	3,029
train	Benzo [a] anthracene	30	6	0,7	1,852	228,3	10	3	2,9	1,911	3,029
train	Benzo [a] anthracene	30	8	0,7	1,973	228,3	10	3	2,9	1,911	3,029
valid	Benzo [a] anthracene	30	4	0,8	1,513	228,3	10	3	2,9	1,911	3,029
train	Benzo [a] anthracene	30	6	0,8	1,673	228,3	10	3	2,9	1,911	3,029
train	Benzo [a] anthracene	30	8	0,8	1,78	228,3	10	3	2,9	1,911	3,029
train	Benzo [a] anthracene	35	4	0,6	1,75	228,3	10	3	2,9	1,911	3,029
train	Benzo [a] anthracene	35	6	0,6	1,934	228,3	10	3	2,9	1,911	3,029
train	Benzo [a] anthracene	35	8	0,6	2,041	228,3	10	3	2,9	1,911	3,029

train	Benzo [a] anthracene	35	4	0,7	1,547	228,3	10	3	2,9	1,911	3,029
train	Benzo [a] anthracene	35	6	0,7	1,704	228,3	10	3	2,9	1,911	3,029
train	Benzo [a] anthracene	35	8	0,7	1,804	228,3	10	3	2,9	1,911	3,029
valid	Benzo [a] anthracene	35	4	0,8	1,388	228,3	10	3	2,9	1,911	3,029
train	Benzo [a] anthracene	35	6	0,8	1,541	228,3	10	3	2,9	1,911	3,029
train	Benzo [a] anthracene	35	8	0,8	1,627	228,3	10	3	2,9	1,911	3,029
train	Benzo [a] anthracene	27,5	5	0,65	1,972	228,3	10	3	2,9	1,911	3,029
train	Benzo [a] anthracene	27,5	7	0,65	2,15	228,3	10	3	2,9	1,911	3,029
train	Benzo [a] anthracene	27,5	5	0,75	1,749	228,3	10	3	2,9	1,911	3,029
train	Benzo [a] anthracene	27,5	7	0,75	1,896	228,3	10	3	2,9	1,911	3,029
valid	Benzo [a] anthracene	32,5	5	0,65	1,829	228,3	10	3	2,9	1,911	3,029
valid	Benzo [a] anthracene	32,5	7	0,65	1,975	228,3	10	3	2,9	1,911	3,029
train	Benzo [a] anthracene	32,5	5	0,75	1,635	228,3	10	3	2,9	1,911	3,029
train	Benzo [a] anthracene	32,5	7	0,75	1,762	228,3	10	3	2,9	1,911	3,029
train	Chrysene	25	4	0,6	2,168	228,3	10	3	2,8	2,94	3,06
train	Chrysene	25	6	0,6	2,467	228,3	10	3	2,8	2,94	3,06
valid	Chrysene	25	8	0,6	2,64	228,3	10	3	2,8	2,94	3,06
train	Chrysene	25	4	0,7	1,958	228,3	10	3	2,8	2,94	3,06
train	Chrysene	25	6	0,7	2,212	228,3	10	3	2,8	2,94	3,06
train	Chrysene	25	8	0,7	2,363	228,3	10	3	2,8	2,94	3,06
valid	Chrysene	25	4	0,8	1,725	228,3	10	3	2,8	2,94	3,06
train	Chrysene	25	6	0,8	1,957	228,3	10	3	2,8	2,94	3,06
valid	Chrysene	25	8	0,8	2,145	228,3	10	3	2,8	2,94	3,06
train	Chrysene	30	4	0,6	2,05	228,3	10	3	2,8	2,94	3,06
train	Chrysene	30	6	0,6	2,278	228,3	10	3	2,8	2,94	3,06
train	Chrysene	30	8	0,6	2,436	228,3	10	3	2,8	2,94	3,06
train	Chrysene	30	4	0,7	1,81	228,3	10	3	2,8	2,94	3,06
train	Chrysene	30	6	0,7	2,004	228,3	10	3	2,8	2,94	3,06
train	Chrysene	30	8	0,7	2,145	228,3	10	3	2,8	2,94	3,06
train	Chrysene	30	4	0,8	1,629	228,3	10	3	2,8	2,94	3,06
valid	Chrysene	30	6	0,8	1,815	228,3	10	3	2,8	2,94	3,06
train	Chrysene	30	8	0,8	1,94	228,3	10	3	2,8	2,94	3,06
train	Chrysene	35	4	0,6	1,865	228,3	10	3	2,8	2,94	3,06
train	Chrysene	35	6	0,6	2,073	228,3	10	3	2,8	2,94	3,06
train	Chrysene	35	8	0,6	2,196	228,3	10	3	2,8	2,94	3,06
train	Chrysene	35	4	0,7	1,651	228,3	10	3	2,8	2,94	3,06
valid	Chrysene	35	6	0,7	1,831	228,3	10	3	2,8	2,94	3,06
train	Chrysene	35	8	0,7	1,947	228,3	10	3	2,8	2,94	3,06
train	Chrysene	35	4	0,8	1,484	228,3	10	3	2,8	2,94	3,06
train	Chrysene	35	6	0,8	1,66	228,3	10	3	2,8	2,94	3,06
train	Chrysene	35	8	0,8	1,761	228,3	10	3	2,8	2,94	3,06
train	Chrysene	27,5	5	0,65	2,13	228,3	10	3	2,8	2,94	3,06
train	Chrysene	27,5	7	0,65	2,338	228,3	10	3	2,8	2,94	3,06
train	Chrysene	27,5	5	0,75	1,894	228,3	10	3	2,8	2,94	3,06
train	Chrysene	27,5	7	0,75	2,066	228,3	10	3	2,8	2,94	3,06
valid	Chrysene	32,5	5	0,65	1,965	228,3	10	3	2,8	2,94	3,06
train	Chrysene	32,5	7	0,65	2,133	228,3	10	3	2,8	2,94	3,06
train	Chrysene	32,5	5	0,75	1,76	228,3	10	3	2,8	2,94	3,06
train	Chrysene	32,5	7	0,75	1,908	228,3	10	3	2,8	2,94	3,06

test	Benzo [b] fluoranthene	25	4	0,6	2,688	252,32	19	2	5,5	1,202	4,557
test	Benzo [b] fluoranthene	25	6	0,6	3,167	252,32	19	2	5,5	1,202	4,557
test	Benzo [b] fluoranthene	25	8	0,6	3,471	252,32	19	2	5,5	1,202	4,557
test	Benzo [b] fluoranthene	25	4	0,7	2,46	252,32	19	2	5,5	1,202	4,557
test	Benzo [b] fluoranthene	25	6	0,7	2,88	252,32	19	2	5,5	1,202	4,557
test	Benzo [b] fluoranthene	25	8	0,7	3,154	252,32	19	2	5,5	1,202	4,557
test	Benzo [b] fluoranthene	25	4	0,8	2,193	252,32	19	2	5,5	1,202	4,557
test	Benzo [b] fluoranthene	25	6	0,8	2,576	252,32	19	2	5,5	1,202	4,557
test	Benzo [b] fluoranthene	25	8	0,8	2,893	252,32	19	2	5,5	1,202	4,557
test	Benzo [b] fluoranthene	30	4	0,6	2,537	252,32	19	2	5,5	1,202	4,557
test	Benzo [b] fluoranthene	30	6	0,6	2,919	252,32	19	2	5,5	1,202	4,557
test	Benzo [b] fluoranthene	30	8	0,6	3,196	252,32	19	2	5,5	1,202	4,557
test	Benzo [b] fluoranthene	30	4	0,7	2,33	252,32	19	2	5,5	1,202	4,557
test	Benzo [b] fluoranthene	30	6	0,7	2,601	252,32	19	2	5,5	1,202	4,557
test	Benzo [b] fluoranthene	30	8	0,7	2,853	252,32	19	2	5,5	1,202	4,557
test	Benzo [b] fluoranthene	30	4	0,8	2,066	252,32	19	2	5,5	1,202	4,557
test	Benzo [b] fluoranthene	30	6	0,8	2,384	252,32	19	2	5,5	1,202	4,557
test	Benzo [b] fluoranthene	30	8	0,8	2,609	252,32	19	2	5,5	1,202	4,557
test	Benzo [b] fluoranthene	35	4	0,6	2,306	252,32	19	2	5,5	1,202	4,557
test	Benzo [b] fluoranthene	35	6	0,6	2,648	252,32	19	2	5,5	1,202	4,557
test	Benzo [b] fluoranthene	35	8	0,6	2,871	252,32	19	2	5,5	1,202	4,557
test	Benzo [b] fluoranthene	35	4	0,7	2,067	252,32	19	2	5,5	1,202	4,557
test	Benzo [b] fluoranthene	35	6	0,7	2,37	252,32	19	2	5,5	1,202	4,557
test	Benzo [b] fluoranthene	35	8	0,7	2,579	252,32	19	2	5,5	1,202	4,557
test	Benzo [b] fluoranthene	35	4	0,8	1,88	252,32	19	2	5,5	1,202	4,557
test	Benzo [b] fluoranthene	35	6	0,8	2,173	252,32	19	2	5,5	1,202	4,557
test	Benzo [b] fluoranthene	35	8	0,8	2,362	252,32	19	2	5,5	1,202	4,557
test	Benzo [b] fluoranthene	27,5	5	0,65	2,705	252,32	19	2	5,5	1,202	4,557
test	Benzo [b] fluoranthene	27,5	7	0,65	3,057	252,32	19	2	5,5	1,202	4,557
test	Benzo [b] fluoranthene	27,5	5	0,75	2,44	252,32	19	2	5,5	1,202	4,557
test	Benzo [b] fluoranthene	27,5	7	0,75	2,736	252,32	19	2	5,5	1,202	4,557
test	Benzo [b] fluoranthene	32,5	5	0,65	2,492	252,32	19	2	5,5	1,202	4,557
test	Benzo [b] fluoranthene	32,5	7	0,65	2,783	252,32	19	2	5,5	1,202	4,557
test	Benzo [b] fluoranthene	32,5	5	0,75	2,261	252,32	19	2	5,5	1,202	4,557
test	Benzo [b] fluoranthene	32,5	7	0,75	2,522	252,32	19	2	5,5	1,202	4,557
valid	Benzo [k] fluoranthene	25	4	0,6	2,983	252,32	18	2	7,3	0,996	4,062
train	Benzo [k] fluoranthene	25	6	0,6	3,569	252,32	18	2	7,3	0,996	4,062
train	Benzo [k] fluoranthene	25	8	0,6	3,958	252,32	18	2	7,3	0,996	4,062
train	Benzo [k] fluoranthene	25	4	0,7	2,744	252,32	18	2	7,3	0,996	4,062
valid	Benzo [k] fluoranthene	25	6	0,7	3,27	252,32	18	2	7,3	0,996	4,062
train	Benzo [k] fluoranthene	25	8	0,7	3,626	252,32	18	2	7,3	0,996	4,062
train	Benzo [k] fluoranthene	25	4	0,8	2,458	252,32	18	2	7,3	0,996	4,062
train	Benzo [k] fluoranthene	25	6	0,8	2,936	252,32	18	2	7,3	0,996	4,062
train	Benzo [k] fluoranthene	25	8	0,8	3,344	252,32	18	2	7,3	0,996	4,062
train	Benzo [k] fluoranthene	30	4	0,6	2,8	252,32	18	2	7,3	0,996	4,062
valid	Benzo [k] fluoranthene	30	6	0,6	3,271	252,32	18	2	7,3	0,996	4,062
train	Benzo [k] fluoranthene	30	8	0,6	3,621	252,32	18	2	7,3	0,996	4,062
train	Benzo [k] fluoranthene	30	4	0,7	2,54	252,32	18	2	7,3	0,996	4,062
train	Benzo [k] fluoranthene	30	6	0,7	2,931	252,32	18	2	7,3	0,996	4,062

train	Benzo [k] fluoranthene	30	8	0,7	3,252	252,32	18	2	7,3	0,996	4,062
train	Benzo [k] fluoranthene	30	4	0,8	2,303	252,32	18	2	7,3	0,996	4,062
train	Benzo [k] fluoranthene	30	6	0,8	2,701	252,32	18	2	7,3	0,996	4,062
train	Benzo [k] fluoranthene	30	8	0,8	2,994	252,32	18	2	7,3	0,996	4,062
valid	Benzo [k] fluoranthene	35	4	0,6	2,53	252,32	18	2	7,3	0,996	4,062
train	Benzo [k] fluoranthene	35	6	0,6	2,945	252,32	18	2	7,3	0,996	4,062
valid	Benzo [k] fluoranthene	35	8	0,6	3,225	252,32	18	2	7,3	0,996	4,062
train	Benzo [k] fluoranthene	35	4	0,7	2,279	252,32	18	2	7,3	0,996	4,062
train	Benzo [k] fluoranthene	35	6	0,7	2,651	252,32	18	2	7,3	0,996	4,062
train	Benzo [k] fluoranthene	35	8	0,7	2,915	252,32	18	2	7,3	0,996	4,062
valid	Benzo [k] fluoranthene	35	4	0,8	2,084	252,32	18	2	7,3	0,996	4,062
train	Benzo [k] fluoranthene	35	6	0,8	2,445	252,32	18	2	7,3	0,996	4,062
train	Benzo [k] fluoranthene	35	8	0,8	2,687	252,32	18	2	7,3	0,996	4,062
train	Benzo [k] fluoranthene	27,5	5	0,65	3,028	252,32	18	2	7,3	0,996	4,062
valid	Benzo [k] fluoranthene	27,5	7	0,65	3,468	252,32	18	2	7,3	0,996	4,062
valid	Benzo [k] fluoranthene	27,5	5	0,75	2,745	252,32	18	2	7,3	0,996	4,062
train	Benzo [k] fluoranthene	27,5	7	0,75	3,122	252,32	18	2	7,3	0,996	4,062
train	Benzo [k] fluoranthene	32,5	5	0,65	2,771	252,32	18	2	7,3	0,996	4,062
valid	Benzo [k] fluoranthene	32,5	7	0,65	3,134	252,32	18	2	7,3	0,996	4,062
train	Benzo [k] fluoranthene	32,5	5	0,75	2,529	252,32	18	2	7,3	0,996	4,062
train	Benzo [k] fluoranthene	32,5	7	0,75	2,858	252,32	18	2	7,3	0,996	4,062
train	Benzo [a]pyrene	25	4	0,6	3,196	252,32	22	6	3,3	2,093	2,714
train	Benzo [a]pyrene	25	6	0,6	3,851	252,32	22	6	3,3	2,093	2,714
valid	Benzo [a]pyrene	25	8	0,6	4,337	252,32	22	6	3,3	2,093	2,714
valid	Benzo [a]pyrene	25	4	0,7	2,946	252,32	22	6	3,3	2,093	2,714
train	Benzo [a]pyrene	25	6	0,7	3,533	252,32	22	6	3,3	2,093	2,714
train	Benzo [a]pyrene	25	8	0,7	3,94	252,32	22	6	3,3	2,093	2,714
train	Benzo [a]pyrene	25	4	0,8	2,651	252,32	22	6	3,3	2,093	2,714
train	Benzo [a]pyrene	25	6	0,8	3,185	252,32	22	6	3,3	2,093	2,714
train	Benzo [a]pyrene	25	8	0,8	3,637	252,32	22	6	3,3	2,093	2,714
valid	Benzo [a]pyrene	30	4	0,6	3,015	252,32	22	6	3,3	2,093	2,714
train	Benzo [a]pyrene	30	6	0,6	3,55	252,32	22	6	3,3	2,093	2,714
train	Benzo [a]pyrene	30	8	0,6	3,955	252,32	22	6	3,3	2,093	2,714
train	Benzo [a]pyrene	30	4	0,7	2,751	252,32	22	6	3,3	2,093	2,714
train	Benzo [a]pyrene	30	6	0,7	3,193	252,32	22	6	3,3	2,093	2,714
train	Benzo [a]pyrene	30	8	0,7	3,561	252,32	22	6	3,3	2,093	2,714
train	Benzo [a]pyrene	30	4	0,8	2,494	252,32	22	6	3,3	2,093	2,714
valid	Benzo [a]pyrene	30	6	0,8	2,945	252,32	22	6	3,3	2,093	2,714
train	Benzo [a]pyrene	30	8	0,8	3,281	252,32	22	6	3,3	2,093	2,714
train	Benzo [a]pyrene	35	4	0,6	2,74	252,32	22	6	3,3	2,093	2,714
train	Benzo [a]pyrene	35	6	0,6	3,217	252,32	22	6	3,3	2,093	2,714
train	Benzo [a]pyrene	35	8	0,6	3,546	252,32	22	6	3,3	2,093	2,714
train	Benzo [a]pyrene	35	4	0,7	2,476	252,32	22	6	3,3	2,093	2,714
train	Benzo [a]pyrene	35	6	0,7	2,905	252,32	22	6	3,3	2,093	2,714
train	Benzo [a]pyrene	35	8	0,7	3,211	252,32	22	6	3,3	2,093	2,714
valid	Benzo [a]pyrene	35	4	0,8	2,27	252,32	22	6	3,3	2,093	2,714
train	Benzo [a]pyrene	35	6	0,8	2,682	252,32	22	6	3,3	2,093	2,714
train	Benzo [a]pyrene	35	8	0,8	2,964	252,32	22	6	3,3	2,093	2,714
valid	Benzo [a]pyrene	27,5	5	0,65	3,269	252,32	22	6	3,3	2,093	2,714

train	Benzo [a]pyrene	27,5	7	0,65	3,767	252,32	22	6	3,3	2,093	2,714
train	Benzo [a]pyrene	27,5	5	0,75	2,971	252,32	22	6	3,3	2,093	2,714
valid	Benzo [a]pyrene	27,5	7	0,75	3,403	252,32	22	6	3,3	2,093	2,714
valid	Benzo [a]pyrene	32,5	5	0,65	3,009	252,32	22	6	3,3	2,093	2,714
valid	Benzo [a]pyrene	32,5	7	0,65	3,427	252,32	22	6	3,3	2,093	2,714
train	Benzo [a]pyrene	32,5	5	0,75	2,753	252,32	22	6	3,3	2,093	2,714
train	Benzo [a]pyrene	32,5	7	0,75	3,132	252,32	22	6	3,3	2,093	2,714
train	Dibenzo [a,h]anthracene	25	4	0,6	3,662	278,36	15	4	6	4,578	3,926
train	Dibenzo [a,h]anthracene	25	6	0,6	4,546	278,36	15	4	6	4,578	3,926
train	Dibenzo [a,h]anthracene	25	8	0,6	5,249	278,36	15	4	6	4,578	3,926
train	Dibenzo [a,h]anthracene	25	4	0,7	3,409	278,36	15	4	6	4,578	3,926
train	Dibenzo [a,h]anthracene	25	6	0,7	4,231	278,36	15	4	6	4,578	3,926
train	Dibenzo [a,h]anthracene	25	8	0,7	4,846	278,36	15	4	6	4,578	3,926
train	Dibenzo [a,h]anthracene	25	4	0,8	3,081	278,36	15	4	6	4,578	3,926
train	Dibenzo [a,h]anthracene	25	6	0,8	3,861	278,36	15	4	6	4,578	3,926
train	Dibenzo [a,h]anthracene	25	8	0,8	4,528	278,36	15	4	6	4,578	3,926
train	Dibenzo [a,h]anthracene	30	4	0,6	3,436	278,36	15	4	6	4,578	3,926
valid	Dibenzo [a,h]anthracene	30	6	0,6	4,182	278,36	15	4	6	4,578	3,926
train	Dibenzo [a,h]anthracene	30	8	0,6	4,773	278,36	15	4	6	4,578	3,926
train	Dibenzo [a,h]anthracene	30	4	0,7	3,181	278,36	15	4	6	4,578	3,926
train	Dibenzo [a,h]anthracene	30	6	0,7	3,814	278,36	15	4	6	4,578	3,926
train	Dibenzo [a,h]anthracene	30	8	0,7	4,362	278,36	15	4	6	4,578	3,926
train	Dibenzo [a,h]anthracene	30	4	0,8	2,911	278,36	15	4	6	4,578	3,926
train	Dibenzo [a,h]anthracene	30	6	0,8	3,562	278,36	15	4	6	4,578	3,926
train	Dibenzo [a,h]anthracene	30	8	0,8	4,071	278,36	15	4	6	4,578	3,926
train	Dibenzo [a,h]anthracene	35	4	0,6	3,119	278,36	15	4	6	4,578	3,926
valid	Dibenzo [a,h]anthracene	35	6	0,6	3,773	278,36	15	4	6	4,578	3,926
valid	Dibenzo [a,h]anthracene	35	8	0,6	4,262	278,36	15	4	6	4,578	3,926
train	Dibenzo [a,h]anthracene	35	4	0,7	2,851	278,36	15	4	6	4,578	3,926
train	Dibenzo [a,h]anthracene	35	6	0,7	3,456	278,36	15	4	6	4,578	3,926
train	Dibenzo [a,h]anthracene	35	8	0,7	3,918	278,36	15	4	6	4,578	3,926
train	Dibenzo [a,h]anthracene	35	4	0,8	2,673	278,36	15	4	6	4,578	3,926
train	Dibenzo [a,h]anthracene	35	6	0,8	3,234	278,36	15	4	6	4,578	3,926
train	Dibenzo [a,h]anthracene	35	8	0,8	3,663	278,36	15	4	6	4,578	3,926
train	Dibenzo [a,h]anthracene	27,5	5	0,65	3,822	278,36	15	4	6	4,578	3,926
train	Dibenzo [a,h]anthracene	27,5	7	0,65	4,534	278,36	15	4	6	4,578	3,926
train	Dibenzo [a,h]anthracene	27,5	5	0,75	3,515	278,36	15	4	6	4,578	3,926
train	Dibenzo [a,h]anthracene	27,5	7	0,75	4,149	278,36	15	4	6	4,578	3,926
train	Dibenzo [a,h]anthracene	32,5	5	0,65	3,508	278,36	15	4	6	4,578	3,926
train	Dibenzo [a,h]anthracene	32,5	7	0,65	4,11	278,36	15	4	6	4,578	3,926
train	Dibenzo [a,h]anthracene	32,5	5	0,75	3,251	278,36	15	4	6	4,578	3,926
valid	Dibenzo [a,h]anthracene	32,5	7	0,75	3,808	278,36	15	4	6	4,578	3,926
train	Benzo[g,h,i] perylene	25	4	0,6	3,775	276,34	47	9	0,1	3,51	3,136
train	Benzo[g,h,i] perylene	25	6	0,6	4,681	276,34	47	9	0,1	3,51	3,136
train	Benzo[g,h,i] perylene	25	8	0,6	5,329	276,34	47	9	0,1	3,51	3,136
train	Benzo[g,h,i] perylene	25	4	0,7	3,519	276,34	47	9	0,1	3,51	3,136
valid	Benzo[g,h,i] perylene	25	6	0,7	4,34	276,34	47	9	0,1	3,51	3,136
train	Benzo[g,h,i] perylene	25	8	0,7	4,948	276,34	47	9	0,1	3,51	3,136
train	Benzo[g,h,i] perylene	25	4	0,8	3,202	276,34	47	9	0,1	3,51	3,136

train	Benzo[g,h,i] perylene	25	6	0,8	3,959	276,34	47	9	0,1	3,51	3,136
train	Benzo[g,h,i] perylene	25	8	0,8	4,611	276,34	47	9	0,1	3,51	3,136
train	Benzo[g,h,i] perylene	30	4	0,6	3,578	276,34	47	9	0,1	3,51	3,136
train	Benzo[g,h,i] perylene	30	6	0,6	4,34	276,34	47	9	0,1	3,51	3,136
train	Benzo[g,h,i] perylene	30	8	0,6	4,939	276,34	47	9	0,1	3,51	3,136
valid	Benzo[g,h,i] perylene	30	4	0,7	3,31	276,34	47	9	0,1	3,51	3,136
train	Benzo[g,h,i] perylene	30	6	0,7	3,951	276,34	47	9	0,1	3,51	3,136
train	Benzo[g,h,i] perylene	30	8	0,7	4,502	276,34	47	9	0,1	3,51	3,136
train	Benzo[g,h,i] perylene	30	4	0,8	3,027	276,34	47	9	0,1	3,51	3,136
train	Benzo[g,h,i] perylene	30	6	0,8	3,68	276,34	47	9	0,1	3,51	3,136
train	Benzo[g,h,i] perylene	30	8	0,8	4,188	276,34	47	9	0,1	3,51	3,136
train	Benzo[g,h,i] perylene	35	4	0,6	3,279	276,34	47	9	0,1	3,51	3,136
train	Benzo[g,h,i] perylene	35	6	0,6	3,956	276,34	47	9	0,1	3,51	3,136
train	Benzo[g,h,i] perylene	35	8	0,6	4,46	276,34	47	9	0,1	3,51	3,136
valid	Benzo[g,h,i] perylene	35	4	0,7	2,996	276,34	47	9	0,1	3,51	3,136
train	Benzo[g,h,i] perylene	35	6	0,7	3,618	276,34	47	9	0,1	3,51	3,136
valid	Benzo[g,h,i] perylene	35	8	0,7	4,087	276,34	47	9	0,1	3,51	3,136
train	Benzo[g,h,i] perylene	35	4	0,8	2,773	276,34	47	9	0,1	3,51	3,136
train	Benzo[g,h,i] perylene	35	6	0,8	3,372	276,34	47	9	0,1	3,51	3,136
train	Benzo[g,h,i] perylene	35	8	0,8	3,805	276,34	47	9	0,1	3,51	3,136
train	Benzo[g,h,i] perylene	27,5	5	0,65	3,953	276,34	47	9	0,1	3,51	3,136
train	Benzo[g,h,i] perylene	27,5	7	0,65	4,665	276,34	47	9	0,1	3,51	3,136
train	Benzo[g,h,i] perylene	27,5	5	0,75	3,634	276,34	47	9	0,1	3,51	3,136
train	Benzo[g,h,i] perylene	27,5	7	0,75	4,264	276,34	47	9	0,1	3,51	3,136
train	Benzo[g,h,i] perylene	32,5	5	0,65	3,661	276,34	47	9	0,1	3,51	3,136
valid	Benzo[g,h,i] perylene	32,5	7	0,65	4,275	276,34	47	9	0,1	3,51	3,136
train	Benzo[g,h,i] perylene	32,5	5	0,75	3,385	276,34	47	9	0,1	3,51	3,136
valid	Benzo[g,h,i] perylene	32,5	7	0,75	3,948	276,34	47	9	0,1	3,51	3,136
valid	Indeno[1,2,3-cd]pyrene	25	4	0,6	3,967	276,34	39	5	6,4	2,051	3,988
train	Indeno[1,2,3-cd]pyrene	25	6	0,6	4,985	276,34	39	5	6,4	2,051	3,988
train	Indeno[1,2,3-cd]pyrene	25	8	0,6	5,73	276,34	39	5	6,4	2,051	3,988
train	Indeno[1,2,3-cd]pyrene	25	4	0,7	3,719	276,34	39	5	6,4	2,051	3,988
train	Indeno[1,2,3-cd]pyrene	25	6	0,7	4,648	276,34	39	5	6,4	2,051	3,988
train	Indeno[1,2,3-cd]pyrene	25	8	0,7	5,353	276,34	39	5	6,4	2,051	3,988
train	Indeno[1,2,3-cd]pyrene	25	4	0,8	3,375	276,34	39	5	6,4	2,051	3,988
train	Indeno[1,2,3-cd]pyrene	25	6	0,8	4,255	276,34	39	5	6,4	2,051	3,988
train	Indeno[1,2,3-cd]pyrene	25	8	0,8	4,825	276,34	39	5	6,4	2,051	3,988
valid	Indeno[1,2,3-cd]pyrene	30	4	0,6	3,748	276,34	39	5	6,4	2,051	3,988
train	Indeno[1,2,3-cd]pyrene	30	6	0,6	4,596	276,34	39	5	6,4	2,051	3,988
train	Indeno[1,2,3-cd]pyrene	30	8	0,6	5,278	276,34	39	5	6,4	2,051	3,988
train	Indeno[1,2,3-cd]pyrene	30	4	0,7	3,508	276,34	39	5	6,4	2,051	3,988
train	Indeno[1,2,3-cd]pyrene	30	6	0,7	4,203	276,34	39	5	6,4	2,051	3,988
train	Indeno[1,2,3-cd]pyrene	30	8	0,7	4,834	276,34	39	5	6,4	2,051	3,988
train	Indeno[1,2,3-cd]pyrene	30	4	0,8	3,194	276,34	39	5	6,4	2,051	3,988
train	Indeno[1,2,3-cd]pyrene	30	6	0,8	3,933	276,34	39	5	6,4	2,051	3,988
train	Indeno[1,2,3-cd]pyrene	30	8	0,8	4,522	276,34	39	5	6,4	2,051	3,988
train	Indeno[1,2,3-cd]pyrene	35	4	0,6	3,414	276,34	39	5	6,4	2,051	3,988
train	Indeno[1,2,3-cd]pyrene	35	6	0,6	4,16	276,34	39	5	6,4	2,051	3,988
train	Indeno[1,2,3-cd]pyrene	35	8	0,6	4,725	276,34	39	5	6,4	2,051	3,988

train	Indeno[1,2,3-cd]pyrene	35	4	0,7	3,132	276,34	39	5	6,4	2,051	3,988
train	Indeno[1,2,3-cd]pyrene	35	6	0,7	3,823	276,34	39	5	6,4	2,051	3,988
train	Indeno[1,2,3-cd]pyrene	35	8	0,7	4,356	276,34	39	5	6,4	2,051	3,988
train	Indeno[1,2,3-cd]pyrene	35	4	0,8	2,911	276,34	39	5	6,4	2,051	3,988
train	Indeno[1,2,3-cd]pyrene	35	6	0,8	3,582	276,34	39	5	6,4	2,051	3,988
train	Indeno[1,2,3-cd]pyrene	35	8	0,8	4,079	276,34	39	5	6,4	2,051	3,988
train	Indeno[1,2,3-cd]pyrene	27,5	5	0,65	4,186	276,34	39	5	6,4	2,051	3,988
train	Indeno[1,2,3-cd]pyrene	27,5	7	0,65	4,993	276,34	39	5	6,4	2,051	3,988
train	Indeno[1,2,3-cd]pyrene	27,5	5	0,75	3,863	276,34	39	5	6,4	2,051	3,988
train	Indeno[1,2,3-cd]pyrene	27,5	7	0,75	4,583	276,34	39	5	6,4	2,051	3,988
train	Indeno[1,2,3-cd]pyrene	32,5	5	0,65	3,855	276,34	39	5	6,4	2,051	3,988
train	Indeno[1,2,3-cd]pyrene	32,5	7	0,65	4,543	276,34	39	5	6,4	2,051	3,988
train	Indeno[1,2,3-cd]pyrene	32,5	5	0,75	3,58	276,34	39	5	6,4	2,051	3,988
train	Indeno[1,2,3-cd]pyrene	32,5	7	0,75	4,219	276,34	39	5	6,4	2,051	3,988
